# Development and Preliminary Characterization of Polyester-Urethane Microparticles Used in Curcumin Drug Delivery System for Oropharyngeal Cancer

**DOI:** 10.3390/medicina58111689

**Published:** 2022-11-21

**Authors:** Alexandru Chioreanu, Ion Cristian Mot, Delia Ioana Horhat, Nicolae Constantin Balica, Cristian Andrei Sarau, Raluca Morar, Eugenia Maria Domuta, Catalin Dumitru, Rodica Anamaria Negrean, Bogdan Andrei Bumbu, Madhavi Ravulapalli, Satish Alambaram, Raja Akshay, Marius Pricop

**Affiliations:** 1 Department of Ear-Nose-Throat, “Victor Babes” University of Medicine and Pharmacy Timisoara, Eftimie Murgu Square 2, 300041 Timisoara, Romania; 2 Surgery Department, Faculty of Medicine and Pharmacy, University of Oradea, Piata 1 Decembrie 10, 410073 Oradea, Romania; 3 Department of Obstetrics and Gynecology, “Victor Babes” University of Medicine and Pharmacy Timisoara, 300041 Timisoara, Romania; 4 Faculty of Medicine and Pharmacy, University of Oradea, 410073 Oradea, Romania; 5 Department of Dental Medicine, Faculty of Medicine and Pharmacy, University of Oradea, 410073 Oradea, Romania; 6 School of General Medicine, Bhaskar Medical College, Amdapur Road 156-162, Hyderabad 500075, India; 7 Malla Reddy Institute of Medical Sciences, Suraram Main Road 138, Hyderabad 500055, India; 8 Discipline of Oral and Maxillo-Facial Surgery, Faculty of Dental Medicine, “Victor Babes” University of Medicine and Pharmacy Timisoara, Eftimie Murgu Square 2, 300041 Timisoara, Romania

**Keywords:** drug delivery systems, polyurethane, cancer management, curcumin, release kinetics

## Abstract

*Background and Objectives*: Curcumin (Cc) as an active substance is known for its anti-inflammatory, anticoagulant, antioxidant, and anti-carcinogenic effects, together with its role in cholesterol regulation, and its use in different gastrointestinal derangements. On the other hand, curcumin can be used for its properties as an inactive substance, with Cc particles being more often tested in pharmaceutical formulations for drug delivery, with promising safety records and kinetics. The aim of this research was to obtain and characterize polyurethane microparticles that can be used as a carrier with a controlled Cc release. *Materials and Methods*: The in vitro samples were characterized by the Zetasizer procedure, and UV–Vis spectroscopy, while the in-vivo measurements on human subjects were performed by non-invasive skin assays (trans-epidermal water loss, erythema, and skin hydration). A total of 16 patients with oropharyngeal cancer stages II and III in equal proportions were recruited for participation. *Results*: The experimental values of sample characteristics using the Zetasizer identified a mean structural size of 215 nm in the polyester-urethane preparate (PU), compared to 271 nm in the curcumin-based PU. Although the size was statistically significantly different, the IPDI and Zeta potential did not differ significantly (22.91 mV vs. 23.74 mV). The average age during the study period was 57.6 years for patients in the PU group, respectively, and 55.1 years in those who received the curcumin preparations. The majority of oropharyngeal cancers were of HPV-related etiology. There were no significant side effects; 75.0% of patients in the PU group reporting no side effects, compared to 87.5% in the Cc group. The 48 h TEWL measurement at the end of the experiment found a statistically significant difference between the PU and the Cc group (2.2 g/h/m^2^ vs. 2.6 g/h/m^2^). The erythema assessment showed a starting measurement point for both research groups with a 5.1-unit difference. After 48 h, the difference between PU and PU_Cc was just 1.7 units (*p*-value = 0.576). The overall difference compared to the reference group with sodium lauryl sulfate (SLS) was statistically significant at a 95% significance level. *Conclusions*: Our findings indicate the obtaining of almost homogeneous particles with a medium tendency to form agglomerations, with a good capacity of encapsulation (around 60%), a medium release rate, and a non-irritative potential. Therefore, this polyester-urethane with Cc microparticles can be tested in other clinical evaluations.

## 1. Introduction

When current pharmaceutical formulations do not offer the essential attributes for a particular medical purpose, drug delivery systems, which are also known as drug carriers, are used in the medical industry. It is possible for these organic or inorganic nano- and micro-structures to encapsulate the active drugs in order to modify their physicochemical properties and to increase drug absorption; by appropriately selecting the delivery system, one can increase the therapeutic effect of the encapsulated drug or can ensure its prolonged or targeted release, thereby avoiding the risks of underdosing or overdosing [1]. It has been shown in the past that altering the polyurethane chains by utilizing diethanolamine as a chain extender results in an improved cytotoxic impact on cancer cells [2]. These findings were compared to those obtained using other diethanolamine polyurethane micelles as a reference. The scientists came to the conclusion that because of this, the structure of the polyurethane carrier has a considerable impact on the loading, pH-triggered release, and intracellular delivery of the medication.

Although the process of drug release using this polyester-urethane delivery system was demonstrated using paclitaxel as the target drug, other non-reactive substances are hypothesized to be used with this method for various cancer histology and different anatomic regions. Around 500,000 people are diagnosed with oral and oropharyngeal cancers each year [3]. This makes the O-OPCs group the sixth most prevalent kind of cancer overall. There has been a concerning rise in the prevalence of O-OPCs in younger patients, notably in Africa (17.2%) and the Middle East (14.5%) [4]. A significant amount of diversity was found in the epidemiology of O-OPCs that was seen all over the globe depending on the geographical distribution, gender, and age of the people. It is possible that the incidence of oropharyngeal cancer is rising among younger age groups in the Western Hemisphere. This may be due to an increased relationship with human papillomavirus 16, which may also be responsible for the rise [5]. 

The general risk factors for oropharyngeal cancers are represented by gender, being twice as common in men, probably due to a higher smoking and alcohol consumption [6], while smoking independently is present in approximately 80% of those with oropharyngeal cancer, and alcohol consumption in 70% of cases [7]. Other important risk factors are patients’ age, with the average onset around 62 years, two-thirds of oropharyngeal cancer patients being older than 55, exposure to UV radiation, poor diet behavior low in fruits and vegetables, and genetic risk factors. Recently, scientific attention has been distributed towards the HPV infection of the oropharyngeal region due to the significant increase in recent years in developed countries [8,9].

Although surgery is used less commonly in the treatment of oropharyngeal cancer, it is an essential component of the management of oral cavity and pharyngeal cancer in terms of the removal of the main tumor and neck lymph nodes. Surgery may be performed alone or in conjunction with radiation, chemotherapy, and immunotherapy/biotherapy to treat early-stage illness. Besides the conventional treatments, other attempts have been made, such as the use of curcumin (turmeric), with a demonstrated potential benefit in various cancers [10]. Although turmeric is often referred to as a single plant, the term defines a genus that includes nearly 100 species of perennials in the Zingiberaceae family [11]. Basically, curcumin, a yellow polyphenol, is the active turmeric ingredient, a plant that began to be used in India 4000 years ago, extracted from the processing of the turmeric rhizome. It reaches turmeric powder in a proportion of 2.5 to 4%. Due to the beneficial properties of curcumin, it can also be found in the composition of food supplements, in a much higher concentration than in powder [12]. Curcumin is a powerful anti-inflammatory agent that acts at the molecular level. Several studies showed that it could block the action of the NF-kB protein complex. Although it plays an important role in the immune system, the irregular action of the NF-kB molecule can lead to viral infections, septic shock, and inflammatory and autoimmune diseases [13].

Although there are studies affirming that curcumin is not a harmful substance to humans, some believe that improper administration can cause side effects such as nausea and diarrhea. On the other hand, curcumin is not recommended for pregnant or breastfeeding women or for patients suffering from kidney or gallbladder stones [14]. It has also been observed that turmeric may have anticoagulant properties, which is why its association with anticoagulant medication may increase the risk of bleeding [15]. Turmeric may also interact with nonsteroidal anti-inflammatory drugs (ibuprofen, naproxen). Besides the many advantages, curcumin has many disadvantages for medical uses, including poor water solubility, limited oral bioavailability, quick liver metabolism, and rapid systemic elimination [16]. Various curcumin encapsulations are used nowadays for effective delivery and a decrease in the needed dosage to achieve the desired effect. Different curcumin encapsulations showed more antioxidant activity than curcumin in its bulk form. Therefore, the main purposes of this research were to obtain a polymer-drug carrier that can deliver curcumin with a specific release rate and determine specific safety features for chemotherapy use in a sample of patients with oropharyngeal cancer.

## 2. Materials and Methods

### 2.1. Design and Ethical Considerations

The current study was designed as an experimental pilot study on human subjects, aiming to determine the characteristics of a drug delivery method using polyester-urethane microparticles for an encapsulated active agent of Curcumin (Cc). The in vitro and in vivo experiments were carried out at the “Victor Babes” University of Medicine and Pharmacy from Timisoara, Romania, following the entire jurisdiction and the principles of the Helsinki Declaration. The experiments were supported by the grant *5EXP/1244/30.01.2020* from “Victor Babes” University of Medicine and Pharmacy, Timisoara, Romania, and approved by the Ethics Committee of the “Victor Babes” University of Medicine and Pharmacy. Sixteen patients suffering from oropharyngeal cancer were recruited to participate in the current study, ingesting the Cc preparations and being assessed by a few non-invasive determinations of skin parameters such as trans-epidermal water loss, erythema, and the stratum corneum hydration have revealed the safe character of these samples to be used on humans.

### 2.2. The Reagents

The raw materials used in the synthesis of the polyester-urethane microparticles (PeU_MP) are 1,4-butanediol (BD) from Carl Roth GmbH (Karlsruhe, Germany), the solvent (acetone) from Chimopar S.A. (Bucuresti, Romania), while the others: polyethylene-glycol (PEG, M ≈ 200), the surfactant (Tween^®^20), isophorone-diisocyanate (IPDI), and hexamethylene-diisocyanate (HMDI) were obtained from Merck (Darmstadt, Germany); polycaprolactone diol (PC), average Mn ~ 530) was achieved from Sigma-Aldrich (St. Louis, MO, USA). All reagents were used as received, without any previous purification. Different salts of Na and K (Cl-, HPO_42_-, H_2_PO_4_- and HCO_3_-) and HCl 1N were obtained from Chimopar S.A. (Bucuresti, Romania), and they were used to obtain a simulated body fluid, which was used as a proper degradation medium for the drug delivery system and its release profile. The drug release medium used was phosphate buffer pH 7.4, 280 mosm/L, 0.13 M.

### 2.3. Chemical Synthesis

**Figure 1:**
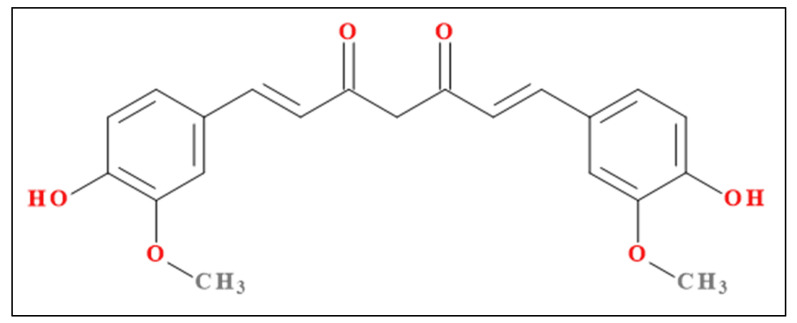
Chemical structure of curcumin (original picture).

A procedure based on different steps was used to synthesize the drug delivery system [17,18,19,20], as follows: (1) An organic component based on a mixture of IPDI and HMDI in acetone (Table 1) was prepared under magnetic stirring (450 rpm) at 25 °C for 10 min; (2) A hydroxylic component based on a mixture of BD, PC, PEG, and Tween^®^20 in deionized water was homogenized (450 rpm) at 25 °C for 10 min; (3) The components were then mixed together (450 rpm) at 30 °C for 3 h due to the absence of any catalyst; (4) The obtained products were repeatedly washed and centrifuged, and they were dried as thin layers in Petri dishes at 65 °C for almost 48 h. The experiment was repeated two times to synthesize a sample with empty particles (PU) and another sample of PeU_MP with Cc (PU_Cc), with the chemical structure presented in Figure 1.

### 2.4. Sample Calibration

**Figure 2:**
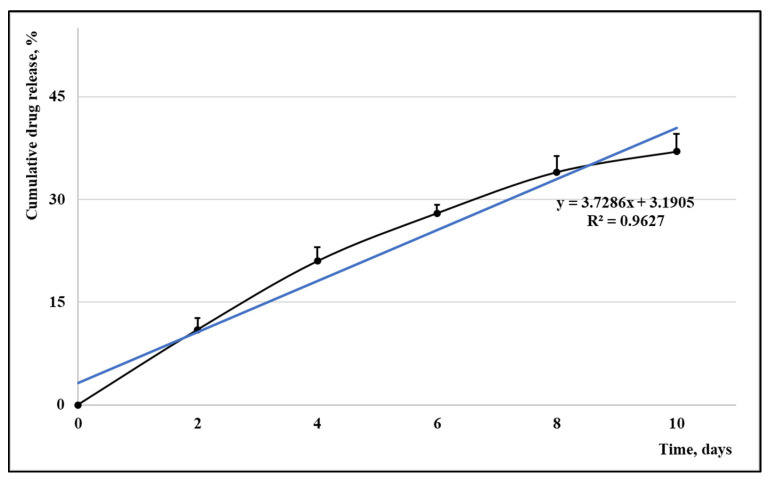
The release profile of Cc from sample PU_Cc.

The percentage of the encapsulated Cc was calculated by reporting the amount of free Cc to its total quantity that was added in that synthesis. An UVi Line 9400 Spectrophotometer (SI Analytics, Mainz, Germany), a calibration curve (Figure 2), and the Beer–Lambert law were involved in establishing the encapsulation percentage. The release rate of the synthesized carrier was evaluated by maintaining the sample PU_Cc inside a degradation medium for ten days; 1 mL medium was replaced every second day with fresh medium, and its absorption was read in triplicate; average values +/− errors were used to present the release profile, as seen in Figure 2. The size and agglomeration tendency of Peu_MP were estimated using a Vasco Particle Size Analyzer and a Wallis Zeta potential Analyzer (Cordouan Technology, Pessac, France); the following parameters were set: assessment temperature (21 ± 1 °C), the interval of time (20 ± 3 µs), number of channels (around 480), power of the laser (85–90%), acquisition mode (continuous), analysis mode (Pade–Laplace), Wallis resolution (medium), and Smoluchowski model as Henry function. The drug entrapment efficiency (DEE) was calculated with the following formula:

DEE: (Total Drug conc. − Supernatant Drug conc.)/(Total Drug conc.) × 100%(1)

### 2.5. Sample Analysis and Statistical Analysis

A fast investigation (6 measurements/48 h) were conducted with an MPA System (Courage and Khazaka, Köln, Germany) equipped with a Tewameter^®^TM300 probe for the evaluation of the trans-epidermal water loss, a Mexameter^®^MX18 probe to assess the erythema level, and a Corneometer^®^CM825 probe for the hydration of stratum corneum. All measurements were performed on the following samples: pure Cc, empty PeU_MP (PU), PU_Cc, and sodium lauryl sulfate (SLS) used as reference. Simple ANOVA was involved in determining whether there are any statistically significant differences between different values of more than two groups vs. SLS; * for *p* ≤ 0.05, ** for *p* ≤ 0.01, and *** for *p* ≤ 0.001). Proportional data calculated using Fisher’s exact test; Data presented using mean and standard error of the mean were compared with the Student’s *t*-test.

## 3. Results

### 3.1. Characterization of the Encapsulated Active Agent

**Figure 3:**
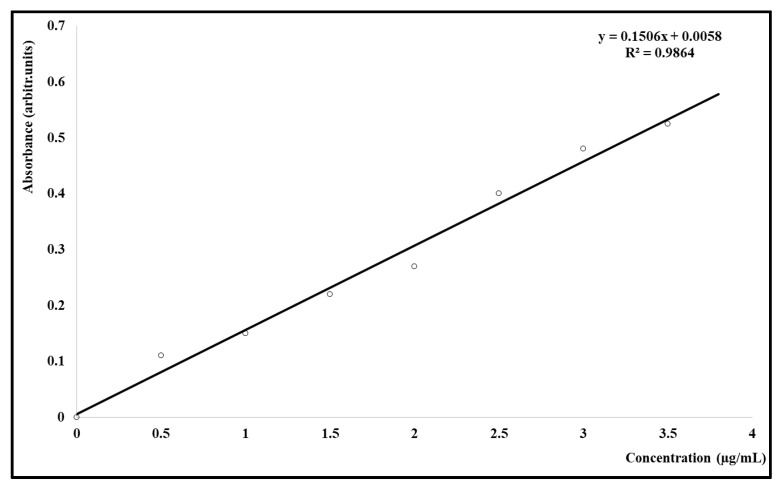
The calibration curve for curcumin (Cc).

A calibration curve between 0 and 3.5 µg/mL Cc (y = 0.1506x + 0.0058; R^2^ = 0.9864) was used to calculate the encapsulation efficiency and the Cc release, as described in Figure 3. A good capacity to encapsulate Cc (61.24%) was found in the case of sample PU_Cc. The drug release trend, expressed as a function of the cumulative drug concentration vs. time, is probably the main parameter that describes a drug delivery system. The drug carriers can be classified into different groups based on their release rate: fast release (a few hours/maximum one day), medium release rate (a few days/maximum one week), and respectively late release (more than ten days). The obtained PeU_MP has a medium release rate.

### 3.2. Characterization of the Zetasizer

Table 2 presents the particle characterization, which was obtained during the Zetasizer measurements. There were obtained microparticles with a medium homogeneity (the polydispersity index, PDI is around 0.5) and a medium tendency to form agglomerations, according to Gallardo et al. [21]. The experimental values of sample characteristics using the Zetasizer identified a mean structural size of 215 nm in the polyester-urethane preparate (PU), compared to 271 nm in the curcumin-based PU (*p*-value < 0.001). Although the size was statistically significantly different, the IPDI and Zeta potential did not differ significantly (22.91 mV vs. 23.74 mV).

### 3.3. In Vivo Characterization of Samples

The irritation assessment is often found in many studies developed on new pharmaceutical formulations. These assays are proper for cosmetics, but many other products can be verified in the same way. Among the study participants (*n* = 16), there group differences between genders did not differ significantly (five men in the PU group vs. four men in the PU_Cc group, *p*-value = 0.614). The average age during the study period was 57.6 years for patients in the PU group, respectively 55.1 years in those who received the curcumin preparations. The majority of oropharyngeal cancers were of HPV-related etiology (75.0% in the PU group vs. 87.5% in the target group, *p*-value = 0.521). Lastly, half of the patients had stage II oropharyngeal cancer on the TNM staging, while the other half were stage III. There were no significant side effects, with 75.0% of patients in the PU group reporting no side effects, compared to 87.5% in the Cc group (*p*-value = 0.583), as described in Table 3.

**Figure 4:**
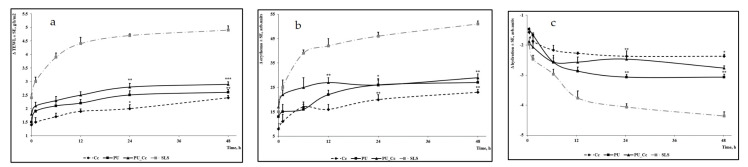
The evolution of the main skin parameters: (**a**) TEWL, (**b**) erythema, (**c**) skin hydration.

Figure 4 and Table 4 present the evolutions of the three skin parameters that present important changes in irritations. As described in Figure 4a, the TEWL measurement at the beginning of the experiment indicated a non-significant difference in the average trans-epidermal water loss (1.9 g/h/m^2^ in the PU group vs. 2.1 g/h/m^2^ in the curcumin group, *p*-value = 0.386). However, the 48 h measurement at the end of the experiment found a statistically significant difference between the PU and the Cc group (2.2 g/h/m^2^ vs. 2.6 g/h/m^2^, *p*-value = 0.013). The overall difference compared to the reference group with sodium lauryl sulfate (SLS) was statistically significant at a 99% significance level.

The erythema evaluation presented in Figure 4b indicated a starting measurement point for both the study groups with a 5.1-unit difference, although without statistical significance (PU = 15.1 units vs. PU_Cc = 20.2 units, *p*-value = 0.223). After 48 h, the difference was reduced to only 1.7 units (PU = 23.4 units vs. PU_Cc = 25.1 units, *p*-value = 0.576). Similar to the TEWL measurement, the comparison with the reference variable (SLS) was statistically significant at the 95% significance level. Lastly, the skin hydration test showed no statistically significant differences between the polyurethane and polyurethane–curcumin group (Figure 4c). The T0 measurement had a starting point at −1.9 units in the PU group and −2.2 in the PU_Cc group (*p*-value = 0.732). The T48 measurement was also non-significant (PU = −3.0 vs. PU_Cc = −2.8, *p*-value = 0.179).

## 4. Discussion

### 4.1. Supporting Literature

The current study describes the main parameters trends of the skin being very similar for the tested samples (pure curcumin, empty polyester-urethane microparticles, PeU_MP with curcumin and sodium lauryl sulfate) but with different amplitudes that favor the curcumin-based drug delivery method. Other researchers have already used sodium lauryl sulfate (SLS) as a reference for measurements of TEWL, erythema, and skin hydration in a 25 h experiment on the skin of healthy human volunteers [22]. It is important to highlight the differences between the values recorded in the case of SLS and the values of samples with Cc for every parameter; the non-irritative potential of these samples is proven by the values of the three parameters, which are only half of the level of the same parameters in the case of SLS.

One of the reasons for developing polyester-urethane with curcumin microparticle drug delivery systems is that other particles, such as Bisphenol-A (BPA), are known to exhibit long-term harmful effects on human health [23]. Multiple investigations have shown that BPA’s hypertensive impact is due to its detrimental influence on endothelial function. In our investigation, we discovered that nanomicelle curcumin produces opposing effects on blood pressure. However, we did not assess its processes. Modulating AT1 receptor (AT1R) expression in arteries and AT1R-mediated vasoconstriction likely reduces the development of hypertension. AT-1 receptor stimulates ROS generation and atherosclerosis development. After activation of angiotensin II (Ang II) by BPA, CaMKIIa was activated, which led to oxidative stress, impaired vascular relaxation, and elevated blood pressure through uncoupling of eNOS [24]. Additionally, BPA was able to activate CaMKIIa through Ang II. Consequently, phospholipase A2 is activated, and arachidonic acid levels rise, causing vasoconstriction and hypertension. Free radicals may promote nitration and inactivation of important enzymes, such as cGMP-dependent protein kinase, the enzyme responsible for NO-mediated vasorelaxation. Endothelial cell activation of endothelin-1 (ET-1) by Ang II, a vasoconstrictor peptide, is another route for BPA-induced hypertension [25]. ET-1 causes vasoconstriction by increasing intracellular calcium61 and decreasing endothelial vasodilation through eNOS deactivation. Therefore, nanomicelle curcumin may have lowered blood pressure through the aforementioned mechanisms.

In one investigation, BPA (50 mg/kg) was shown to increase the phosphorylation of p38 and JNK while lowering the phosphorylation of AKT and ERK1/2. However, nanomicelle curcumin (50 mg/kg) in cardiac tissue enhanced the protein levels of p-AKT and p-ERK while decreasing the protein levels of p-P38 and p-JNK [26]. In contrast to these findings, research indicated that curcumin induces apoptosis in H9c2 cardiac myoblast cells via boosting ROS production and JNK activation [27]. In addition, other research shows that BPA promoted the growth of MCF-7 human breast cancer cells through estrogen receptors and dysregulated p-AKT expression. Curcumin, in contrast, reduced the proliferation-promoting effects of BPA on MCF-7 cells [28]. In other research, 100 nM BPA treatment dramatically increased oxidative stress and activation of JNK, p38, and ERK in HepG2 cells. While curcumin reduced the effects of BPA, it had a greater impact on ERK [29]. 

In other experimental studies, Cc was used with polycarbonate products, where it was demonstrated how the distinctive attributes of Cc would have a favorable impact on the dynamics of the polycarbonate delivery products [30]. Previous research has shown, without a doubt, that curcumin’s antioxidant and biocidal qualities may be put to use in the area of polymers in order to improve the features of such materials. It has been demonstrated that curcumin is a more effective melt and thermo-oxidative stabilizer of polyethylene than the synthetic phenolic antioxidant and that curcumin can be advantageously used in the synthesis of rigid polyurethane foams with enhanced mechanical, antibacterial, and anti-aging properties [31]. In particular, it has been shown that curcumin is a more efficient melt and thermo-oxidative stabilizer of polyethylene than the synthetic phenolic antioxidant, while it is anticipated that the incorporation of Cc into the production of polycarbonate may bring about benefits, particularly in terms of the antioxidant characteristics that they possess, even if more steps and more in-depth characterizations are certainly required.

Studies showed that it is possible to use Cc’s or the derivatives as compounds ready to use in drug delivery systems, increasing the potential of replacing molecules of fossil origin that are concerning for their toxic and eco-toxic effects exhibited. Curcumin products had been successfully implemented recently in various treatment methods for cancer, proving their feasibility for mass production [32]. Similar to our study, it was previously attempted to observe the potential adjuvant effect of curcumin nanoparticles in oral cancer treatment [33]. The researchers added 200 nm nanocurcumin particles in addition to cetuximab for malignant cells expressing KB 3-1 and compared the compound with a reference treatment of cetuximab only. The results were promising, since relative to the cetuximab-alone treatment group, the combined therapy group saw a considerable increase in cell death, while nanocurcumin sensitization significantly increased cell death compared to the control group. Even though the industrial scalability of the proposed synthesis is complicated at the present time by the higher cost of Cc, which is currently being marketed as a food-grade or research-grade material, we are convinced that under the pressure of international legislation and the increased environmental sensitivity of consumers, natural alternatives such as Cc may become accessible also to the polymer industry, which would make a positive development for the environment. Additionally, the EU has imposed severe restrictions on the use of molecules that have been shown to be carcinogenic and endocrine disruptors, such as bisphenol-A, while simultaneously encouraging the search for safer and more environmentally friendly alternatives [34]. 

### 4.2. Study Limitations and Future Perspectives

The current study provides important data about the kinetics, stability, and safety of curcumin-based polyurethane products for chemotherapy use. However, the feasibility of the large-scale use of these products in pharmacology and oncology is yet to be determined. Similarly, the human safety record is promising, but as a pilot study, the number of subjects involved was only sixteen participants; therefore, further trials should be implemented to draw clear conclusions. From a future perspective, greater production and use of non-edible turmeric products might, in the not-too-distant future, result in a dramatic reduction in the cost of industrially used curcumin. In addition, meeting the ecological requirements may be accomplished by combining the production of bio-based polymers with the recycling of commonly generated wastes made of plastic.

## 5. Conclusions

In conclusion, a different preparation was developed in order to mitigate the drawbacks of polyurethane-based medication delivery methods in oncology while simultaneously increasing their therapeutic potential against HCC while maintaining a good safety profile. This paper describes the obtaining and the preliminary characterization of a synthetic drug delivery system used as a carrier for curcumin. Polyester-urethane microparticles with sizes between 215 and 271 nm have been developed, and their release rate was investigated in a simulated body fluid used as a degradation media. Additionally, the safety concerns were tested on patients suffering from oropharyngeal cancer, with very promising results, as the non-invasive determinations of skin parameters such as trans-epidermal water loss, erythema, and stratum corneum hydration have revealed the safe character of these samples to be used on humans. Despite the fact that further study is undoubtedly required, it is possible that this research will prove to be of significant relevance to the field of contemporary polyurethane-based methods for drug delivery.

## Figures and Tables

**Table 1:** The ratios between the raw materials.

Sample	Hydroxylic Comp. (mL/50 mL)				Curcumin (mg/50 mL)	Organic Comp. (mL/50 mL)	
	BD	PC	PEG	Tween		IPDI	HMDI
PU	0.40	0.60	2.15	1.50	0.00	2.15	2.85
PU_Cc	0.40	0.60	2.15	1.50	5.50	2.15	2.85

PU—Empty Particles; PU_Cc—Curcumin-filled Particles; BD—1,4-butanediol; PC—polycaprolactone diol; PEG—polyethylene-glycol; IPDI—isophorone-diisocyanate; HMDI—hexamethylene-diisocyanate.

**Table 2:** Experimental values of the Zetasizer characterization.

Sample	Size of Structures (nm)		Zeta Potential (mV)
	Mean ± SD	IPDI	
PU	215 ± 11	0.4	+22.91
PU_Cc	271 ± 19	0.6	+23.74

PU—Empty Particles; PU_Cc—Curcumin-filled Particles; SD—Standard Deviation; IPDI—isophorone-diisocyanate.

**Table 3:** Background characteristics and medication dynamics among study participants.

Variables	PU (*n* = 8)	PU_Cc (*n* = 8)	*p*-Value
**Gender**			0.614
Men	5 (62.5%)	4 (50.0%)	
Women	3 (37.5%)	4 (50.0%)	
**Age (mean ± SD)**	57.6 ± 12.2	55.1 ± 10.9	0.672
**Etiology**			0.521
HPV-related	6 (75.0%)	7 (87.5%)	
Non-HPV	2 (25.0%)	1 (12.5%)	
**TNM Staging**			1
Stage II	4 (50.0%)	4 (50.0%)	
Stage III	4 (50.0%)	4 (50.0%)	
**Self-reported side effects**			0.583
0	6 (75.0%)	7 (87.5%)	
1	1 (12.5%)	1 (12.5%)	
≥2	1 (12.5%)	0 (0.0%)	

Proportional data calculated using Fisher’s exact test; Data presented using mean and standard deviation were compared with the Student’s *t*-test; SD—Standard deviation; HPV—Human Papilloma Virus; TNM—Tumor Nodes Metastasis cancer staging system.

**Table 4:** Medication dynamics measured among study groups.

Variables (Mean ± SE)	PU (*n* = 8)	PU_Cc (*n* = 8)	*p*-Value
**TEWL**			
Time 0	1.9 ± 0.1	2.1 ± 0.2	0.386
Time 12	2.0 ± 0.2	2.4 ± 0.2	0.179
Time 24	2.2 ± 0.1	2.5 ± 0.2	0.201
Time 48	2.2 ± 0.1	2.6 ± 0.1	0.013
**Erythema**			
Time 0	15.1 ± 3.2	20.2 ± 2.4	0.223
Time 12	21.0 ± 1.9	24.3 ± 3.1	0.379
Time 24	20.3 ± 2.7	20.5 ± 2.9	0.960
Time 48	23.4 ± 2.0	25.1 ± 2.2	0.576
**Skin hydration**			
Time 0	−1.9 ± 0.5	−2.2 ± 0.7	0.732
Time 12	−3.0 ± 0.3	−2.6 ± 0.5	0.503
Time 24	−3.1 ± 0.2	−2.2 ± 0.4	0.063
Time 48	−3.0 ± 0.1	−2.8 ± 0.1	0.179

Data presented using mean and standard error of the mean were compared with the Student’s *t*-test; SE—Standard error; PU—Polyurethane; PU_Cc–Polyurethane–Curcumin; TEWL—Trans-epidermal Water Loss.

## Data Availability

Data available on request.
